# Differentiating early Parkinson’s disease from multiple system atrophy on multimodal PET/MRI using atlas-guided segmentation and vision transformer classification

**DOI:** 10.3389/fnagi.2026.1928959

**Published:** 2026-09-25

**Authors:** Chao Cong, Xuewei Zhang, Shams Tabrez Rifat, Hongyun Tan, Jinju Sun, Wenping Peng, Xiao Chen

**Affiliations:** 1 School of Electrical and Electronic Engineering, Chongqing University of Technology, Chongqing, China; 2 Department of Nuclear Medicine, Daping Hospital, Army Medical University, Chongqing, China; 3 Chongqing Clinical Research Center for Imaging and Nuclear Medicine, Chongqing, China

**Keywords:** computer assisted diagnosis, deep learning, multimodal imaging, multiple system atrophy, Parkinson’s disease

## Abstract

**Introduction:**

Early differentiation between Parkinson’s disease (PD) and multiple system atrophy-parkinsonian type (MSA-P) remains clinically challenging because of overlapping motor symptoms and subtle imaging abnormalities.

**Methods:**

We developed a fully automated multimodal PET/MRI framework for early PD–MSA-P differentiation that integrates atlas-guided subject-specific label generation, nnU-Net segmentation of the caudate nuclei and putamina, multimodal image fusion, and Vision Transformer classification with multi-slice voting (ViT+MSV). A total of 155 early-stage patients, including 121 with PD and 34 with MSA-P, were evaluated using patient-level stratified 4-fold cross-validation.

**Results:**

The segmentation model achieved an overall Dice score of 0.853 (95% CI, 0.830–0.877) and an IoU of 0.747 (95% CI, 0.725–0.770). ViT+MSV achieved a cross-validated AUC of 0.947 (95% CI, 0.905–0.981), an accuracy of 0.929 (95% CI, 0.890–0.968), a sensitivity of 0.942 (95% CI, 0.900–0.982), and a specificity of 0.882 (95% CI, 0.765–0.974). The combination of [^18^F]fluorodeoxyglucose (FDG) PET, [^11^C]CFT dopamine-transporter PET, and T2-weighted imaging (T2WI) showed the best overall classification performance. Grad-CAM visualizations showed that class-discriminative gradient-based saliency was mainly concentrated on the bilateral putamen.

**Discussion:**

These findings suggest that anatomically guided multimodal PET/MRI analysis with transformer-based classification may support early PD–MSA-P differentiation while providing spatially interpretable evidence for model decisions.

## 1 Introduction

Parkinson’s disease (PD) and multiple system atrophy (MSA) are two distinct neurodegenerative diseases that may exhibit similar clinical manifestations in their early stages, making differentiation between them particularly challenging (Poewe et al., 2022). This is especially true for the parkinsonian subtype of MSA (MSA-P), which shares the cardinal features of bradykinesia and rigidity with PD and is therefore the most clinically ambiguous subtype to distinguish from PD in the first years after symptom onset (Krismer et al., 2024). Despite these similarities, PD and MSA-P differ substantially in their underlying pathophysiology, therapeutic responsiveness, and prognostic trajectories, underscoring the importance of accurate early differential diagnosis.

Magnetic Resonance Imaging (MRI) and Positron Emission Tomography (PET) are both widely used to distinguish PD from MSA-P. On conventional MRI, MSA-P may show putaminal atrophy, a decreased putamen-to-caudate volume ratio, and signal changes such as the “putaminal cleft sign,” whereas PD patients typically show no significant striatal atrophy or signal abnormalities, with morphometric indices generally within normal ranges (Li et al., 2023; Gibbons et al., 2023; Bae et al., 2021; Chen et al., 2022). Functional and molecular PET imaging is often more informative at early stages: [^18^F]FDG reveals reduced glucose metabolism in the basal ganglia of MSA-P patients (Fallahpoor et al., 2024; Jost et al., 2025), while dopamine transporter (DAT) tracers reflect the density of presynaptic dopaminergic neurons, whose striatal reduction follows a characteristic pattern in PD that differs from that in MSA-P (Smith et al., 2023; Zarkali et al., 2024). However, these imaging findings are conventionally assessed through visual inspection or handcrafted measurements, which are subjective, experience-dependent, and often limited in sensitivity at the earliest disease stages—motivating the development of automated, quantitative approaches.

Along this direction, a growing body of work has applied machine learning and deep learning (DL) to differentiate PD from atypical parkinsonian syndromes (Krismer et al., 2024; Shokrpour et al., 2025). Earlier studies established the feasibility of MRI-based classification by radiomics methods (Tupe-Waghmare et al., 2021), and CNN-based pipelines (Camacho et al., 2023). More recent work has extended these approaches. Huang et al. (2025) developed a multicenter T1WI/T2WI radiomics model for distinguishing idiopathic PD from MSA-P, although the pipeline remained dependent on handcrafted radiomic features and subsequent feature selection. Recent 3D CNN studies have integrated clinical information with T1-weighted MRI or combined gray-matter-density and mean-diffusivity maps (Basaia et al., 2025; Mattia et al., 2025). In a prospective multicenter study, Vaillancourt et al. (2025) combined quantitative diffusion MRI features with support vector machine learning and achieved strong pairwise discrimination among PD, MSA-P, and PSP. However, the framework remained MRI-only and relied on predefined regional diffusion features rather than direct multimodal image-level representation learning (Vaillancourt et al., 2025). Region-based U-Nets have also been adapted for Parkinson-plus syndromes, enabling accurate segmentation of multiple deep-brain structures (Li et al., 2025). Collectively, these studies established the feasibility of image-based automated differentiation, but many remained MRI-focused or relied on handcrafted or precomputed quantitative representations.

Because MRI alone does not directly characterize metabolic and dopaminergic alterations, multimodal analysis is important for capturing the complementary pathophysiological differences between PD and MSA-P (Cheng et al., 2025; Chen et al., 2023). Earlier multimodal PET/MRI studies of PD–MSA differentiation, including our previous work, primarily relied on radiomics and conventional machine-learning models trained on handcrafted features (Sun et al., 2024). More recently, Chang et al. (2025) combined multimodal and multisequence PET/MR images using a ResNet18 model and aggregated predictions from key axial, coronal, and sagittal slices by majority voting, supporting the complementary value of PET and MRI. Li et al. (2026) further developed a radiomics-guided dual-channel network that combined local features extracted by dilated convolutions with Transformer-based global representations for interpretable classification of IPD, MSA, and PSP using [^18^F]FDG PET. Although the model was validated in an independent external cohort, it remained based on a single PET tracer and did not integrate dopaminergic PET with structural MRI or patient-specific striatal localization (Li et al., 2026). In DL models, the Vision Transformer (ViT) has recently gained traction in medical imaging for its ability to capture long-range dependencies and integrate heterogeneous information across modalities. Unlike conventional CNNs, which are constrained by local receptive fields, ViT leverages self-attention to model complex spatial and contextual interactions, making it particularly suited for multimodal fusion tasks (Raghu et al., 2021; Manzari et al., 2023). Such properties make Transformer-based models particularly attractive for multimodal neuroimaging integration, which is central to this study.

Taken together, recent studies establish the feasibility of automated imaging-based differentiation of parkinsonian syndromes, but three issues remain insufficiently addressed in the specific setting of early PD–MSA-P. First, studies specifically restricted to early-stage PD and MSA-P remain relatively scarce, as recent models have often included broader parkinsonian groups, healthy controls, or mixed disease stages. Second, although multimodal PET/MR and interpretable PET-based deep-learning approaches have emerged, the integration of dual-tracer PET and routine structural MRI with patient-specific striatal localization in a unified image-level framework has received limited attention. Third, existing slice-based PET/MR approaches have generally relied on predefined multiplanar positions and hard majority voting, whereas transparent aggregation of anatomically centered adjacent ROI slices into a continuous patient-level score remains insufficiently explored. Addressing these gaps is clinically relevant because early PD–MSA-P differentiation is made at the patient level, when conventional structural abnormalities may still be subtle. These gaps motivated the present study.

To address these gaps, we developed an automated DL-based framework to differentiate PD and MSA-P by integrating multimodal MRI (T1WI, T2WI, and T2-FLAIR), and PET ([11C]CFT and [18F]FDG) images. This approach is underpinned by the fact that these neurodegenerative disorders exhibit both structural and functional changes that are distinctly observable in the basal ganglia, particularly the caudate nucleus and putamen (Kaasinen et al., 2021; Chung et al., 2022). To analyze these key regions, our framework first employs a two-stage registration process to automatically generate subject-specific segmentation labels from the neuromorphometric atlas. The framework then utilizes nnU-Net model for three-dimensional striatal segmentation (Isensee et al., 2021), followed by fixed-channel image-level PET/MRI fusion, ViT classification, and explicit patient-level multi-slice aggregation. In contrast to approaches based primarily on a single modality, handcrafted radiomic features, or an implicit transition from slice-level outputs to a patient-level decision, the present framework combines structural, metabolic, and dopaminergic information with anatomically constrained segmentation and gradient-based saliency visualization in one workflow.

The main contributions of this work include:

(a). A focus on a clinically challenging cohort of early-stage patients (≤5 years from symptom onset), addressing a critical diagnostic gap.
(b). An automated segmentation pipeline that uses atlas-label transformations to train a robust nnU-Net model, enabling end-to-end extraction of clinically relevant basal ganglia regions.
(c). A ViT-based classification framework with multi-slice voting mechanism for the classification of fused multimodal PET/MRI data, demonstrating a robust approach to integrating complex structural and functional information.
(d). Grad-CAM analysis providing class-specific gradient-based saliency maps in anatomically relevant regions.

## 2 Materials and methods

### 2.1 Patients

The protocol for this retrospective study was approved by the institutional ethics committee of Daping Hospital, Army Medical University (Approval No. 2022157), and the requirement for individual informed consent was waived. All procedures were performed in accordance with the principles of the Declaration of Helsinki. A total of 332 patients with suspected parkinsonian syndromes underwent both PET and MRI examinations.

The inclusion criteria were as follows: (1) clinical diagnosis of either PD or MSA made by experienced neurologists based on the Movement Disorder Society criteria for PD and the Second Consensus Statement for MSA; all enrolled MSA patients were of the parkinsonian subtype. (2) disease duration ≤ 5 years (defined as the time from onset of initial motor symptoms to the date of PET/MRI imaging); (3) availability of both PET ([^18^F]FDG and [^11^C]CFT) and MRI (T1WI, T2WI, T2-FLAIR) scans performed within a 2-week interval; and (4) a clinical follow-up duration of at least 2 years to confirm diagnostic stability.

The exclusion criteria were: (1) failure to meet established diagnostic criteria for PD or MSA-P (*n* = 68); (2) history of other neurological or psychiatric conditions, (e.g., stroke, traumatic brain injury, or mental illness; *n* = 33); (3) disease duration > 5 years (*n* = 45); (4) an interval between PET and MRI exceeding 2 weeks (*n* = 23); and (5) poor image quality or incomplete imaging data (*n* = 8).

Table 1 summarizes the demographic and clinical characteristics of the enrolled patients. Among the 155 participants, 121 were diagnosed with PD and 34 with MSA-P. There were no statistically significant differences between the two groups in terms of age, age at onset, disease duration, CFT_SUVmax, or FDG_SUVmax (all *p* > 0.05).

**TABLE 1:** Baseline demographic and clinical characteristics of enrolled patients.

Clinical features	MSA-P (*n* = 34)	PD (*n* = 121)	*P*-value
Age	59.0 (50.0, 66.5)	60.0 (54.0, 70.5)	0.184
Gender			
Male	16 (47.06%)	75 (61.98%)	0.118
Female	18 (52.94%)	46 (38.02%)	
Age at onset	58.0 (46.8, 62.8)	59.0 (53.0, 69.0)	0.095
Disease duration	2.00 (0.86, 3.00)	1.00 (0.95, 2.00)	0.241
CFT_SUVmax	6.60 ± 1.66	6.21 ± 2.09	0.416
FDG_SUVmax	8.33 ± 2.59	8.65 ± 2.71	0.607

### 2.2 Imaging protocols

Imaging was performed using a 3T Siemens MRI scanner with a 16-element head matrix coil and a Siemens Biograph 64 HD PET/CT scanner. MRI parameters included a matrix size of 290 × 320 for T1WI, 348 × 384 for T2WI, and 224 × 256 for FLAIR; field of view (FOV) was consistently set at 230 × 230 mm for T1WI and T2WI, and 230 × 230 mm for FLAIR, with a slice thickness of 5 mm and 22–23 slices each. PET scans used a matrix of 168 × 168, FOV of 342 × 342 mm, and a slice thickness of 1 mm with 148 slices for both [^18^F]FDG and [^11^C]CFT. The time interval between PET and MRI scans did not exceed 2 weeks, and between FDG and CFT within 1 week for consistency. All imaging data used were acquired in routine clinical practice settings. Detailed scanning parameters are listed in Table 2.

**TABLE 2:** Magnetic Resonance Imaging (MRI) and Positron Emission Tomography (PET) protocol acquisition parameters.

Parameters	T1WI	T2WI	FLAIR	^18^F-FDG	^11^C-CFT
Scanner	3T Siemens	3T Siemens	3T Siemens	Biograph 64 HD (Siemens)	Biograph 64 HD (Siemens)
TR/TE (ms)	250/2.7	4,900/92	8,000/94	–	–
Matrix size (width × height, pixel)	290 × 320	348 × 384	224 × 256	168 × 168	168 × 168
FOV (width × height, mm)	230 × 230	230 × 230	230 × 230	342 × 342	342 × 342
Slice thickness (mm)	5	5	5	1	1
Intersection gap (mm)	1	1	1	–	–
Slice number	22∼23	22∼23	22∼23	148	148
Other parameters	16-channel head coil	Turbo spin-echo	–	Injected dose: 150–200 MBq; fasting > 6 h; scan at 45–60 min post-injection	Injected dose: 350–400 MBq; fasting > 6 h; scan at 45–60 min post-injection

For model development and evaluation, a patient-level stratified 4-fold cross-validation strategy was adopted to provide a more stable estimate of model performance in this relatively small and imbalanced cohort. All images and all selected slices from a given patient were confined to the same fold to prevent patient- and slice-level data leakage. 4-fold were selected *a priori* as a pragmatic balance between training-set size, validation-set stability, and computational cost. With 34 patients with MSA-P, each held-out fold contained approximately 38–39 patients, including approximately 8–9 patients with MSA-P, while approximately 75% of the cohort remained available for training. The validation predictions from all 4-fold were then pooled to obtain the overall cross-validated performance of the proposed framework and all comparative models. Stratification was used to limit distortion of the class distribution across folds, and class-specific sensitivity and specificity with bootstrap confidence intervals were reported so that performance was not judged from accuracy alone. For the reported classification experiments, preprocessing consisted of deterministic resizing and ImageNet normalization; no additional stochastic geometric or intensity augmentation, oversampling, weighted sampling, or class-weighted classification loss was applied. Standard online augmentation configured by nnU-Net was restricted to segmentation training and was not used as disease-class rebalancing.

### 2.3 Algorithm workflow

The four-panel workflow is illustrated in Figure 1. Figure 1A depicts T1WI-referenced within-subject registration and inverse mapping of MNI152 Neuromorphometrics atlas labels to native T1WI space. Figure 1B shows MRI-only 3D nnU-Net segmentation of the bilateral caudate nuclei and putamina. Figure 1C shows common ROI extraction and fixed-channel multimodal stacking. Figure 1D shows slice-wise inference using the same trained ViT on five selected axial slices, followed by fixed weighted-soft multi-slice voting (MSV) to obtain one patient-level decision. Class-specific Grad-CAM was used to visualize spatial attribution for individual fused slices.

**FIGURE 1:**
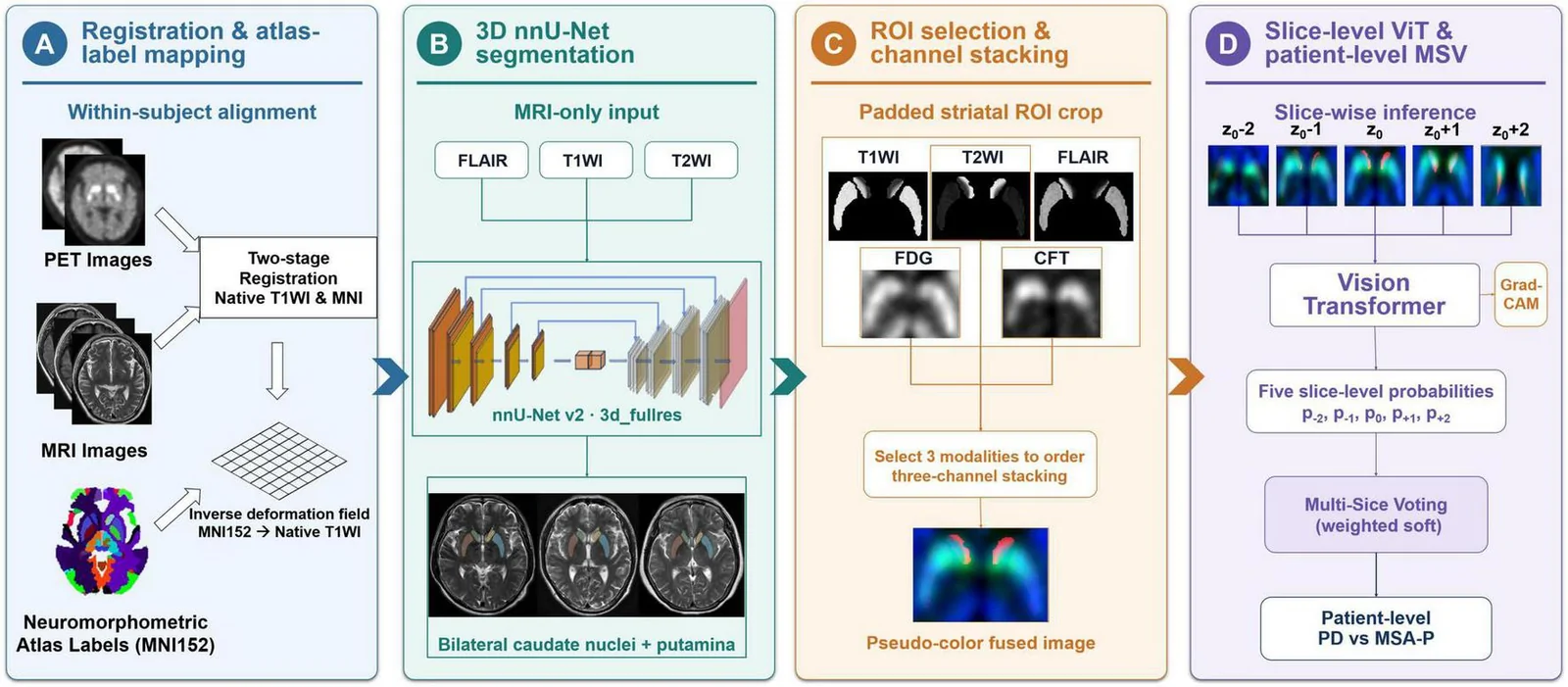
Overview of the multimodal Positron Emission Tomography/Magnetic Resonance Imaging (PET/MRI) framework. **(A)** Within-subject MRI and PET alignment used T1WI as the reference, followed by inverse transformation of MNI152 Neuromorphometrics atlas labels to native T1WI space. **(B)** Co-registered FLAIR, T1WI, and T2WI were used as the three nnU-Net inputs for segmentation of the bilateral caudate nuclei and putamina; PET was not used for segmentation. **(C)** A common striatal ROI was applied across aligned modalities, and three selected grayscale images from the same patient and slice position were stacked in a fixed channel order to form each pseudo-color classifier input. **(D)** Five consecutive axial ROI slices were classified by a Vision Transformer (ViT) model. Slice-level softmax probabilities were combined by fixed weighted soft multi-slice voting (MSV) using weights 0.10, 0.20, 0.40, 0.20, and 0.10 to obtain the patient-level decision. Grad-CAM was used for class-specific gradient attribution.

All model training and inference procedures were performed on an Ubuntu 20.04 workstation equipped with an NVIDIA RTX 3080 GPU (12 GB memory) and Intel Core i7-10700K CPU (3.80 GHz). PyTorch 1.13.1 and CUDA 11.6 were utilized within a Python 3.9 environment.

#### 2.3.1 Two-stage registration

A two-stage registration process was applied to accurately align MRI and PET images, using T1WI as the reference modality (Sun et al., 2024). First, coarse registration was performed with 6 degrees of freedom (DOF) to correct large misalignments. Subsequently, a fine registration with 12 DOF was executed to refine alignment accuracy. Additionally, the inverse deformation field between the native T1WI space and MNI152 standard space was calculated. Neuromorphometric labels (provided by Neuromorphometrics, Inc.)^1^ were registered and transformed to each patient’s native T1WI space using this inverse deformation field, aligning them precisely with the T1WI images. This alignment facilitated the generation of accurate segmentation labels for the putamina and caudate nuclei from the neuromorphometric labels, essential for training the deep learning model.

The registration of these multi-modal sequences was conducted using the 3D Slicer software (Slicer, v4.11^2^), while the MNI deformation pipeline was performed by the Statistical Parametric Mapping 12 tool (SPM12^3^).

#### 2.3.2 Segmentation

The segmentation targeted the dorsal striatum, comprising the caudate nucleus and putamen, which are the region’s most consistently affected by nigrostriatal degeneration in PD and MSA-P. The ventral striatum (nucleus accumbens) was not modeled separately, as the neuromorphometric atlas labels delineate the caudate and putamen as the principal striatal structures relevant to motor parkinsonism.

Co-registered T1WI, T2WI, and FLAIR modalities were used as the three input channels; PET was not used for segmentation. Segmentation was implemented using nnU-Net v2 with the 3d_fullres configuration and the automatically configured PlainConvUNet architecture (Isensee et al., 2021). The volumes were resampled to a common grid of 512 × 512 × 20 by linear interpolation before being input into the nnU-Net model for automated segmentation of the caudate nuclei and putamina. The 3D encoder progressively extracted multiscale features, the decoder restored spatial resolution, and skip connections transferred localized anatomical information between corresponding stages. The network output comprised background and four foreground classes corresponding to the left caudate nucleus, right caudate nucleus, left putamen, and right putamen.

Ground-truth labels were derived by transforming the MNI152 Neuromorphometrics atlas labels to each patient’s native T1WI space using the subject-specific inverse deformation fields. Images and segmentation masks were anonymized, reviewed, and manually corrected by a nuclear medicine physician with over 5 years of clinical experience to ensure anatomical accuracy.

The segmentation model was trained with a batch size of 2 for 150 epochs, using an initial learning rate of 0.001. A composite loss function combining Dice loss and cross-entropy was employed. Standard online spatial and intensity augmentation configured by nnU-Net was applied during segmentation training. Then, the segmentation performance was evaluated by Dice score, Intersection over Union (IoU), sensitivity (Sens), and positive predictive value (PPV), within the patient-level stratified 4-fold cross-validation framework as described in 2.2. For each fold, the nnU-Net model was trained on the three training folds and evaluated only on the held-out validation fold. Dice, IoU, Sens, and PPV were calculated from the pooled out-of-fold results. Importantly, the out-of-fold segmentation predicted for each held-out patient was used for subsequent ROI localization and slice selection. The manually reviewed reference masks were used only for segmentation supervision and evaluation and were not used to construct classifier inputs for held-out patients. Dice score, intersection over union, sensitivity, and positive predictive value were calculated from the pooled out-of-fold predictions.

#### 2.3.3 ROI and fusion

After the segmentation, regions of interest (ROIs) were extracted by computing the smallest bounding rectangle encompassing each ROI and expanding it outward by five pixels to capture segmentation boundaries and minimize edge artifacts. The same bounding-box coordinates were applied to all co-registered modalities from the same patient and axial position. For MRI, only pixels within segmented masks were retained to emphasize anatomical structures; for PET, the entire expanded bounding box content was retained to preserve metabolic context.

For each patient, the axial slice containing the largest area of the combined striatal mask was defined as *z_0_*. The two immediately inferior and two immediately superior slices were selected together with *z_0_*, yielding five subject-specific slice positions: *z_0–2_*, *z_0–1_*, *z_0_*, *z_0_+1*, and *z_0_+2*. Following multimodal co-registration, the corresponding anatomical positions were extracted from all MRI and PET modalities. Thus, the same anatomical locations, rather than the same original scanner slice indices, were used across modalities. All extracted slices were resized to 384 × 384 pixels to standardize inputs for classification.

For each patient and processed slice position, the co-registered T1WI, T2WI, FLAIR, CFT, and FDG images had identical spatial dimensions. The corresponding grayscale images from each modality were assigned to a fixed channel order and stacked to form one three-channel pseudo-color fused image. The channel assignment was held constant within a modality-combination experiment and across cross-validation folds (the detailed channel assignment was recorded in Supplementary Table 1). Figures 1C and 1D illustrate the spatial correspondence, ROI cropping, and channel-stacking procedure.

#### 2.3.4 Classification

A Vision Transformer (ViT-B/16), pretrained on ImageNet-21k, was fine-tuned for two-class classification (PD/MSA-P) on the fused pseudo-color images. Each image was divided into non-overlapping 16 × 16-pixel patches, converted to patch embeddings with positional information and a classification token, processed by Transformer encoder blocks containing multi-head self-attention and multilayer perceptrons, and mapped to PD and MSA-P logits through a linear classification head. Training used a batch size of 2, 150 epochs, and cross-entropy loss. The same trained ViT was applied independently to the five selected slices from each patient, yielding five slice-level softmax probabilities for subsequent MSV aggregation.

Patient-level MSV was implemented as fixed weighted soft aggregation:


$$P_{M\,S\,V}=0.10\,p_{-2}+0.20\,p_{-1}+0.40\,p_{0}+0.20\,p_{+1}+0.10\,p_{+2}\;$$ (1)

The central position received the greatest weight, with symmetrically decreasing weights for neighboring slices. The weights were prespecified based on the anatomical rationale that the central slice contained the largest segmented striatal area and were not optimized using held-out test predictions. A patient was classified as PD when *P*_*MSV*_ > 0.50 and as MSA-P otherwise. The resulting model is denoted ViT+MSV.

For benchmarking, four additional deep learning models were trained under identical conditions: a CNN baseline (ResNet50), CNN+LSTM, a hybrid CNN+ViT, and the standalone ViT without multi-slice voting. In addition, to benchmark the proposed framework against a conventional non-deep-learning approach, we re-evaluated the multimodal PET/MRI radiomics pipeline from our previous work (Sun et al., 2024) on the same cohort under the identical cross-validation protocol. All models were evaluated using the same patient-level stratified 4-fold cross-validation. Classification performance was quantified by accuracy (ACC), sensitivity (Sens), specificity (Spec), and the area under the receiver operating characteristic curve (AUC).

For visual interpretation, Grad-CAM (Selvaraju et al., 2017) was applied to individual fused pseudo-color slices to assess whether class-discriminative saliency was concentrated on clinically relevant regions, particularly the putamen, and to facilitate interpretation of modality-specific contributions to diagnostic decisions. For the visualizations presented in Figures 5, 6, Grad-CAM was computed with respect to the PD output logit.

#### 2.3.5 Statistical analysis

All statistical analyses were performed using SPSS (version 26.0; IBM Corp., Armonk, NY, USA), R (version 4.2.1; R Foundation for Statistical Computing, Vienna, Austria), and MedCalc (version 20.022; MedCalc Software Ltd., Ostend, Belgium). Continuous variables were reported as mean ± standard deviation (SD) or median with interquartile range (IQR), and categorical variables as counts and percentages.

All performance estimates were derived from the pooled out-of-fold predictions obtained through patient-level stratified 4-fold cross-validation, so that every patient contributed exactly once to the evaluation. For MSV, the continuous patient-level score *P*_*MSV*_was used for ROC analysis, AUC estimation, and DeLong comparison. The thresholded patient-level label was used to calculate accuracy, sensitivity, and specificity. Ninety-five percent confidence intervals (95% CIs) for all classification and segmentation metrics were obtained by bootstrap resampling of the pooled out-of-fold predictions with 2,000 replicates. Pairwise AUC comparisons between competing models were assessed using the DeLong test. A two-sided *p*-value < 0.05 was considered statistically significant.

## 3 Results

### 3.1 Segmentation results

The nnU-Net achieved robust cross-validated segmentation performance, with an overall Dice score of 0.853 (95% CI, [0.830–0.877]) and an IoU of 0.747 (95% CI, [0.725–0.770]) across all regions (Table 3). Among the segmented regions, the right caudate nucleus showed the best performance (Dice = 0.884), whereas the right putamen showed comparatively lower performance (Dice = 0.825). Figure 2 illustrates representative segmentation results for three patients, showing the original MRI scans together with the reference labels and predicted segmentations. Overall, the predicted segmentations remained closely aligned with the reference regions, supporting the use of the segmentation outputs for subsequent classification analysis.

**TABLE 3:** Cross-validated segmentation performance across different regions of interest.

Segmentation regions	Dice [95% CI]	Sens [95% CI]	PPV [95% CI]	IoU [95% CI]
Left caudate nucleus	0.871 [0.860–0.882]	0.840 [0.825–0.855]	0.854 [0.839–0.868]	0.742 [0.731–0.753]
Right caudate nucleus	0.884 [0.874–0.894]	0.870 [0.856–0.883]	0.863 [0.850–0.876]	0.788 [0.778–0.798]
Left putamen	0.847 [0.836–0.858]	0.830 [0.815–0.844]	0.856 [0.842–0.869]	0.717 [0.706–0.728]
Right putamen	0.825 [0.813–0.837]	0.800 [0.785–0.814]	0.848 [0.833–0.862]	0.701 [0.689–0.713]
Overall regions	0.853 [0.830–0.877]	0.835 [0.822–0.847]	0.855 [0.843–0.867]	0.747 [0.725–0.770]

**FIGURE 2:**
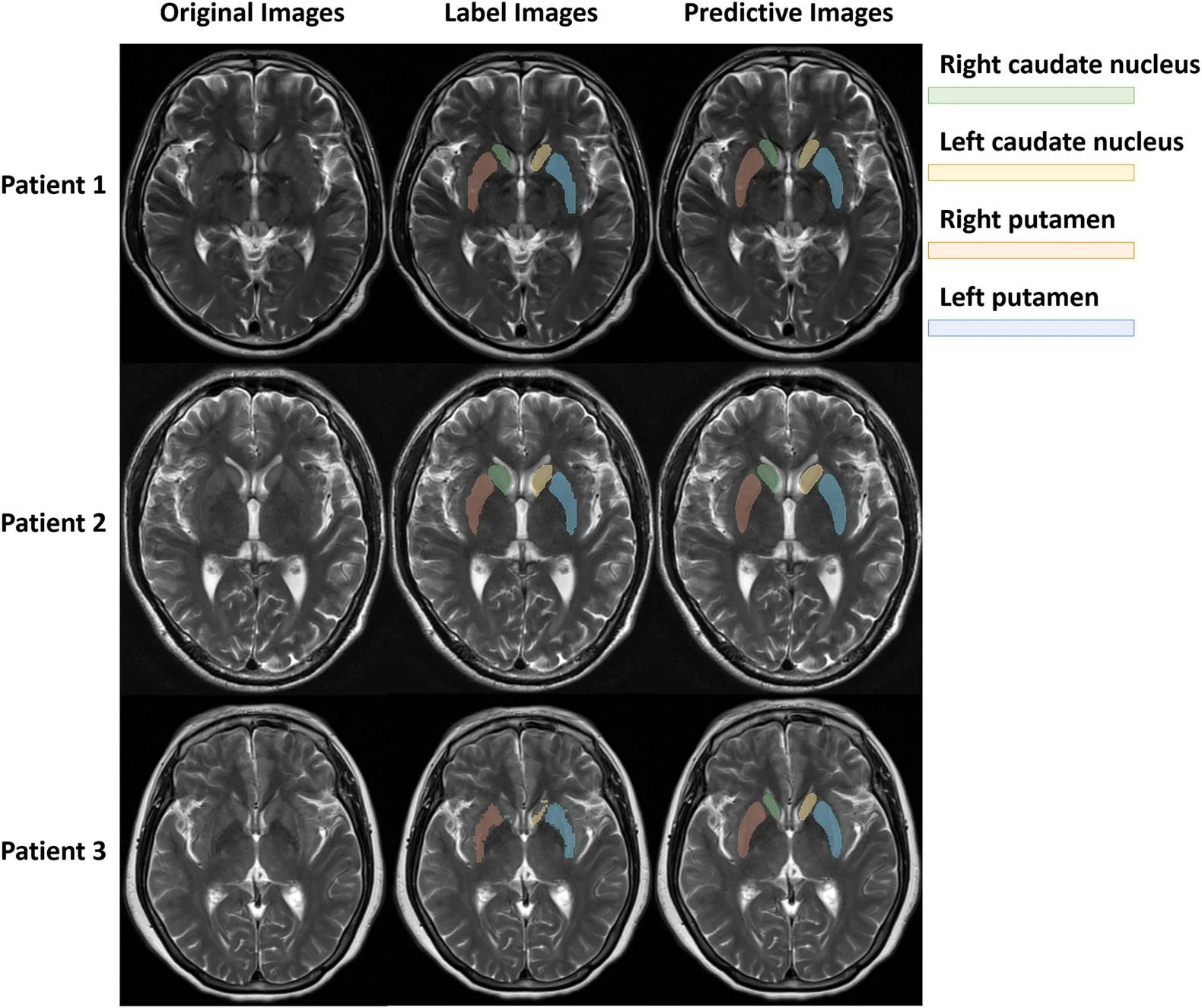
Representative segmentation results for three patients. From left to right: original Magnetic Resonance Imaging (MRI) images, reference labels, and nnU-Net predictions for the bilateral caudate nuclei and putamina.

Minor irregularities were observed in some reference label boundaries, likely reflecting imperfections in atlas-based label generation, as shown in columns 2 and 3 for Patient 3. Nevertheless, the predicted segmentations remained closely aligned with the reference regions overall, supporting their use for downstream classification.

### 3.2 Classification results

The cross-validated classification performance of the five deep learning models together with the radiomics baseline is summarized in Table 4 and visualized in Figure 3. The proposed ViT+MSV model achieved the best overall performance, with an AUC of 0.947 (95% CI, 0.905–0.981), an accuracy of 0.929 (95% CI, 0.890–0.968), a sensitivity of 0.942 (95% CI, 0.900–0.982), and a specificity of 0.882 (95% CI, 0.765–0.974). It outperformed the standalone ViT (AUC 0.845; ΔAUC = +0.102), the hybrid CNN+ViT (AUC 0.805; ΔAUC = +0.142), CNN+LSTM (AUC 0.811; ΔAUC = +0.136), and the CNN baseline (AUC 0.759; ΔAUC = +0.188) (all *P* < 0.001). The multi-slice voting strategy yielded a consistent improvement over the standalone ViT, indicating that aggregating predictions across adjacent slices reduces slice-level variability and stabilizes the patient-level decision. At the patient level, the pooled out-of-fold predictions correctly classified 114 of 121 patients with PD and 30 of 34 patients with MSA-P, corresponding to a balanced accuracy of 0.912. This indicates that the high overall accuracy was not driven solely by predictions favoring the majority PD class.

**TABLE 4:** Cross-validated performance of five deep learning models and the radiomics baseline

Model	AUC [95% CI]	ACC [95% CI]	Sens [95% CI]	Spec [95% CI]	*N* patients
ViT+MSV^*1^	0.947 [0.905–0.981]	0.929 [0.890–0.968]	0.942 [0.900–0.982]	0.882 [0.765–0.974]	155
CNN+ViT	0.805 [0.689–0.905]	0.884 [0.832–0.929]	0.926 [0.874–0.969]	0.735 [0.576–0.882]	155
ViT	0.845 [0.753–0.924]	0.897 [0.852–0.942]	0.950 [0.907–0.984]	0.706 [0.552–0.853]	155
CNN+LSTM	0.811 [0.725–0.887]	0.813 [0.755–0.871]	0.868 [0.808–0.925]	0.618 [0.452–0.778]	155
CNN^*2^	0.759 [0.649–0.852]	0.813 [0.748–0.871]	0.868 [0.808–0.926]	0.618 [0.441–0.781]	155
Radiomics^*3^	0.834 [0.757–0.904]	0.839 [0.774–0.897]	0.835 [0.765–0.898]	0.853 [0.724–0.967]	155

AUC, area under the curve; CI, confidence interval. All metrics were computed from pooled out-of-fold predictions of patient-level stratified 4-fold cross-validation (*n* = 155); 95% CIs were estimated by bootstrap resampling (2,000 replicates). ^*1^ViT+MSV, Vision Transformer with multi-slice voting. ^*2^CNN, convolutional neural network (ResNet50). ^*3^The radiomics model (Sun et al., 2024) denotes the multimodal PET/MRI radiomics approach of our previous work, re-implemented and re-evaluated on the present cohort under the identical cross-validation protocol.

**FIGURE 3:**
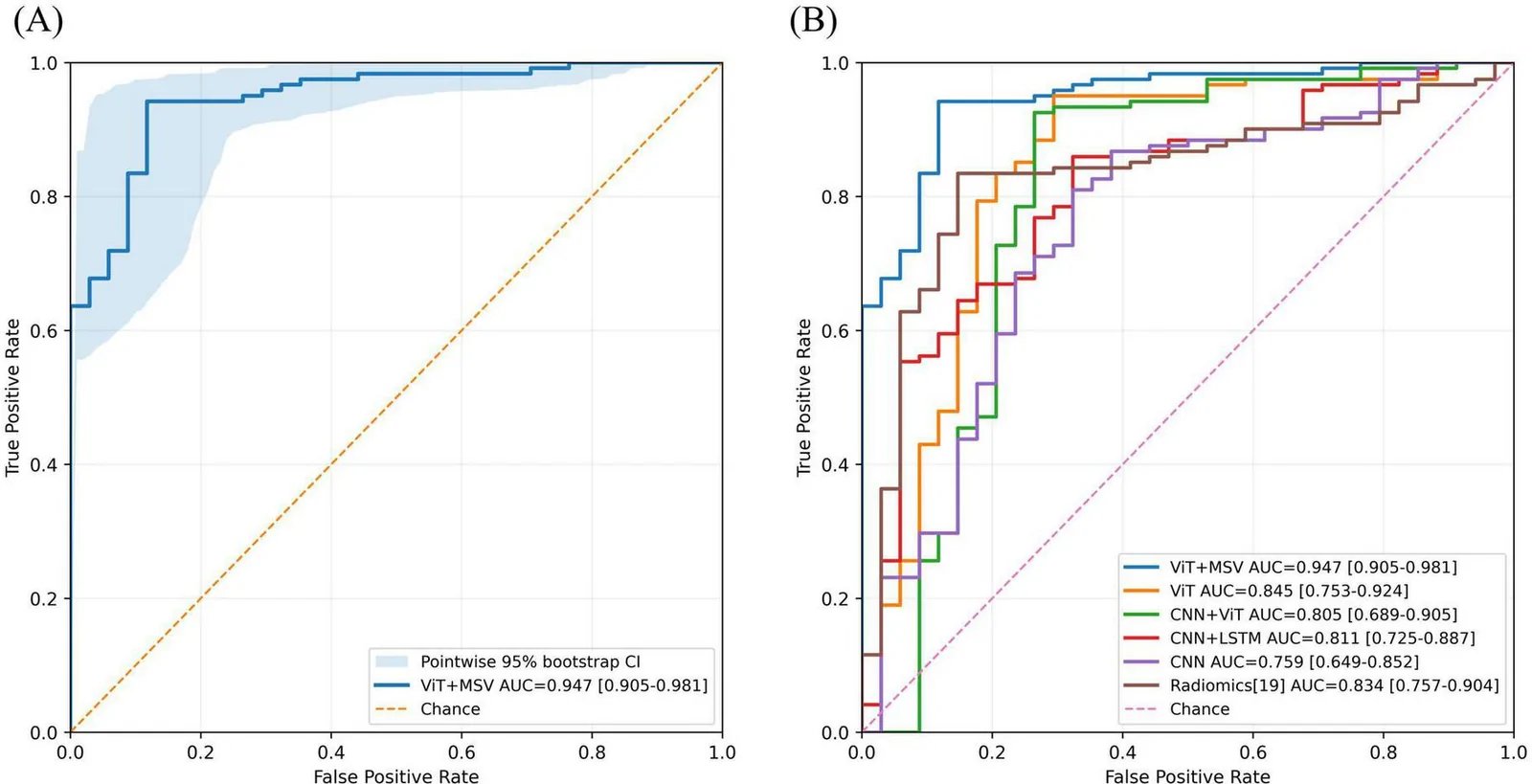
Cross-validated receiver operating characteristic (ROC) curves for model comparison based on pooled patient-level out-of-fold predictions. **(A)** ROC curve of the proposed Vision Transformer with multi-slice voting (ViT+MSV) model, with the pointwise 95% bootstrap confidence band shown in blue. **(B)** ROC curves of ViT+MSV, standalone ViT, CNN+ViT, CNN+LSTM, ResNet50, and the radiomics baseline. AUC, area under the curve; CI, confidence interval.

When benchmarked against the conventional multimodal radiomics approach of the previous work (Sun et al., 2024), re-evaluated on the same cohort under the identical cross-validation protocol, ViT+MSV showed a clear advantage (radiomics AUC 0.834, 95% CI 0.757–0.904; ΔAUC = +0.113, *P* < 0.001). Notably, the re-evaluated radiomics model (AUC 0.834) was consistent with the imaging-model AUC of 0.845 originally reported in previous work (Sun et al., 2024), confirming the fidelity of the re-implementation and supporting the validity of this head-to-head comparison.

All three-modality combinations were evaluated and selected clinically representative combinations are shown in Table 5 and visualized in Figure 4 (complete results of all combinations are provided in Supplementary Table 1). Among all combinations, FDG+CFT+T2WI yielded the highest cross-validated performance (AUC 0.947, 95% CI 0.905–0.981; accuracy 0.929; sensitivity 0.942; specificity 0.882), closely followed by the other tri-modal PET+MRI combinations FDG+CFT+T1WI (AUC 0.929) and FDG+CFT+FLAIR (AUC 0.904). Multimodal fusion consistently outperformed single-modality inputs: the best combination improved on the strongest single modality, FDG alone (AUC 0.859; ΔAUC = +0.088, *P* = 0.001), and substantially exceeded the all-MRI combination T1WI+T2WI+FLAIR (AUC 0.764; ΔAUC = +0.183, *P* < 0.001) and the weakest single sequence, FLAIR (AUC 0.698; ΔAUC = +0.249, *P* < 0.001). These results indicate that combining metabolic (FDG), dopaminergic (CFT), and structural (T2WI) information provides complementary diagnostic value for early PD–MSA-P differentiation, whereas structural MRI sequences alone are insufficient.

**TABLE 5:** Cross-validated performance of selected imaging sequence combinations.

Modality(s)	AUC [95% CI]	ACC [95% CI]	Sens [95% CI]	Spec [95% CI]
FLAIR	0.698 [0.581–0.805]	0.697 [0.626–0.768]	0.669 [0.587–0.752]	0.794 [0.645–0.923]
CFT^*1^	0.840 [0.752–0.917]	0.858 [0.800–0.910]	0.876 [0.816–0.934]	0.794 [0.645–0.923]
FDG^*1^	0.859 [0.789–0.925]	0.858 [0.800–0.910]	0.868 [0.805–0.926]	0.824 [0.684–0.946]
T1WI+T2WI+ FLAIR	0.764 [0.674–0.843]	0.787 [0.723–0.845]	0.785 [0.711–0.854]	0.794 [0.645–0.923]
FDG+CFT+ FLAIR	0.904 [0.831–0.964]	0.929 [0.884–0.968]	0.934 [0.884–0.975]	0.912 [0.800–1.000]
FDG+CFT+T1WI	0.929 [0.879–0.971]	0.929 [0.884–0.968]	0.926 [0.875–0.968]	0.941 [0.853–1.000]
FDG+CFT+T2WI	0.947 [0.905–0.981]	0.929 [0.890–0.968]	0.942 [0.900–0.982]	0.882 [0.765–0.974]

^*1^Single-tracer Positron Emission Tomography (PET) classifier-input ablations under Magnetic Resonance Imaging (MRI)-guided anatomical localization.

**FIGURE 4:**
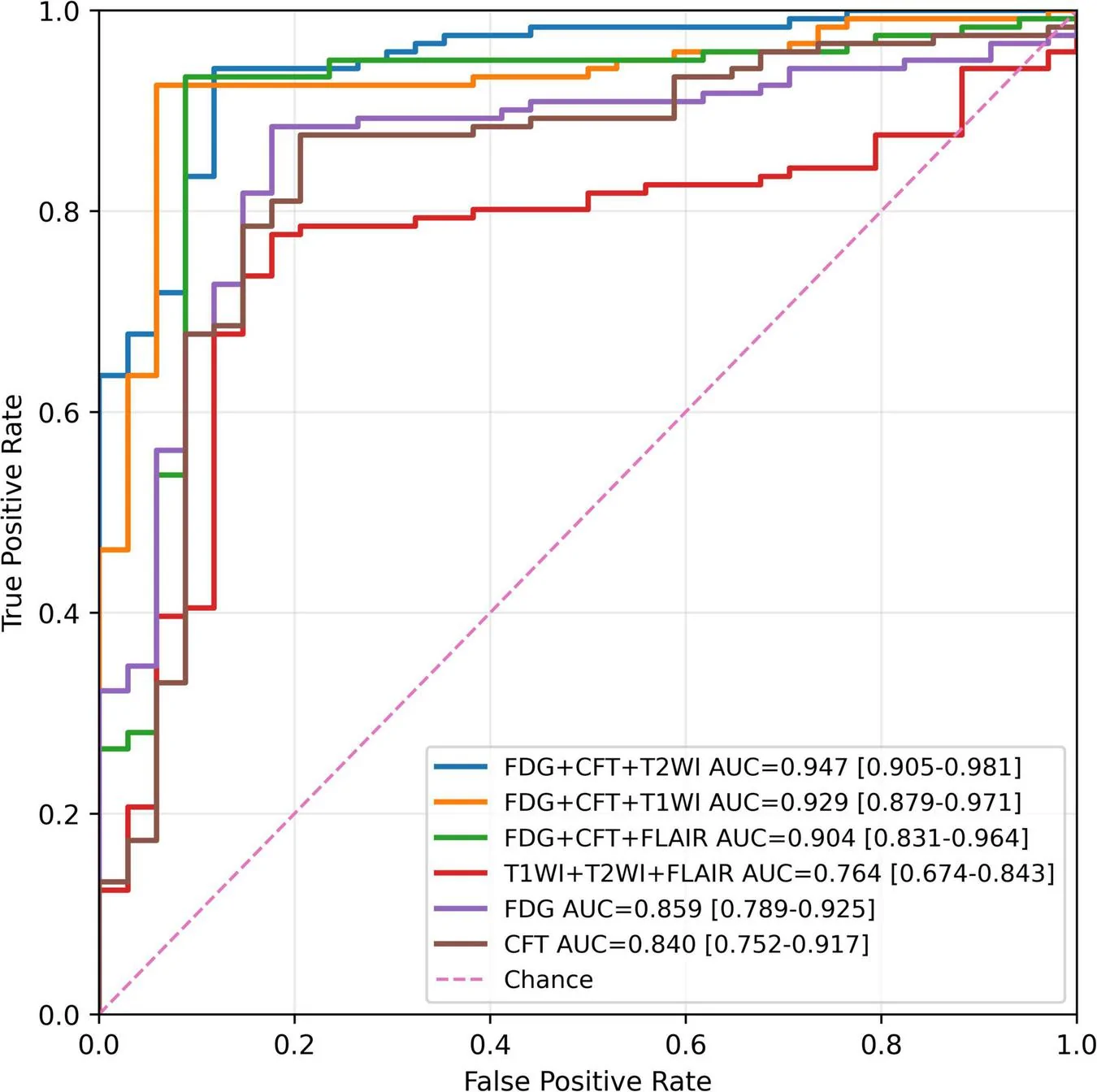
Cross-validated ROC curves of the Vision Transformer with multi-slice voting (ViT+MSV) model using different imaging sequence combinations for differentiating PD from MSA-P. ROC, receiver operating characteristic; AUC, area under the curve.

The modality-specific experiments were conducted as classifier-input ablations to quantify the discriminative contribution of reduced modality sets. The MRI-only T1WI+T2WI+FLAIR model achieved an AUC of 0.764, whereas the single-tracer CFT and FDG inputs achieved AUCs of 0.840 and 0.859, respectively. For the single-tracer PET experiments, only the corresponding PET tracer was used as the classifier input, while anatomical localization and slice selection were still based on the MRI-derived striatal segmentation. These results therefore reflect the discriminative value of individual PET tracers within the present anatomically guided framework.

### 3.3 Grad-CAM visualization

The fused images and corresponding Grad-CAM visualizations generated using the ViT+MSV method are displayed in Figure 5, providing a detailed examination of the model’s performance across different fused images (T1WI+T2WI+FLAIR, CFT+T1WI+T2WI, FDG+T1WI+T2WI, and FDG+CFT+T2WI) for four distinct patients. High activation was consistently observed in the bilateral putamen, especially dorsolateral regions. Correct predictions (patients 1 and 2) aligned with disease-specific markers. For patients 3 and 4, misidentifications were noted with the T1WI+T2WI+FLAIR combination. These cases revealed limitations in certain modality combinations. However, with the CFT+T1WI+T2WI, FDG+T1WI+T2WI, and FDG+CFT+T2WI combinations in the same patients, the model successfully distinguished PD from MSA-P, suggesting that the inclusion of complementary PET information improved discrimination in these representative ambiguous cases.

**FIGURE 5:**
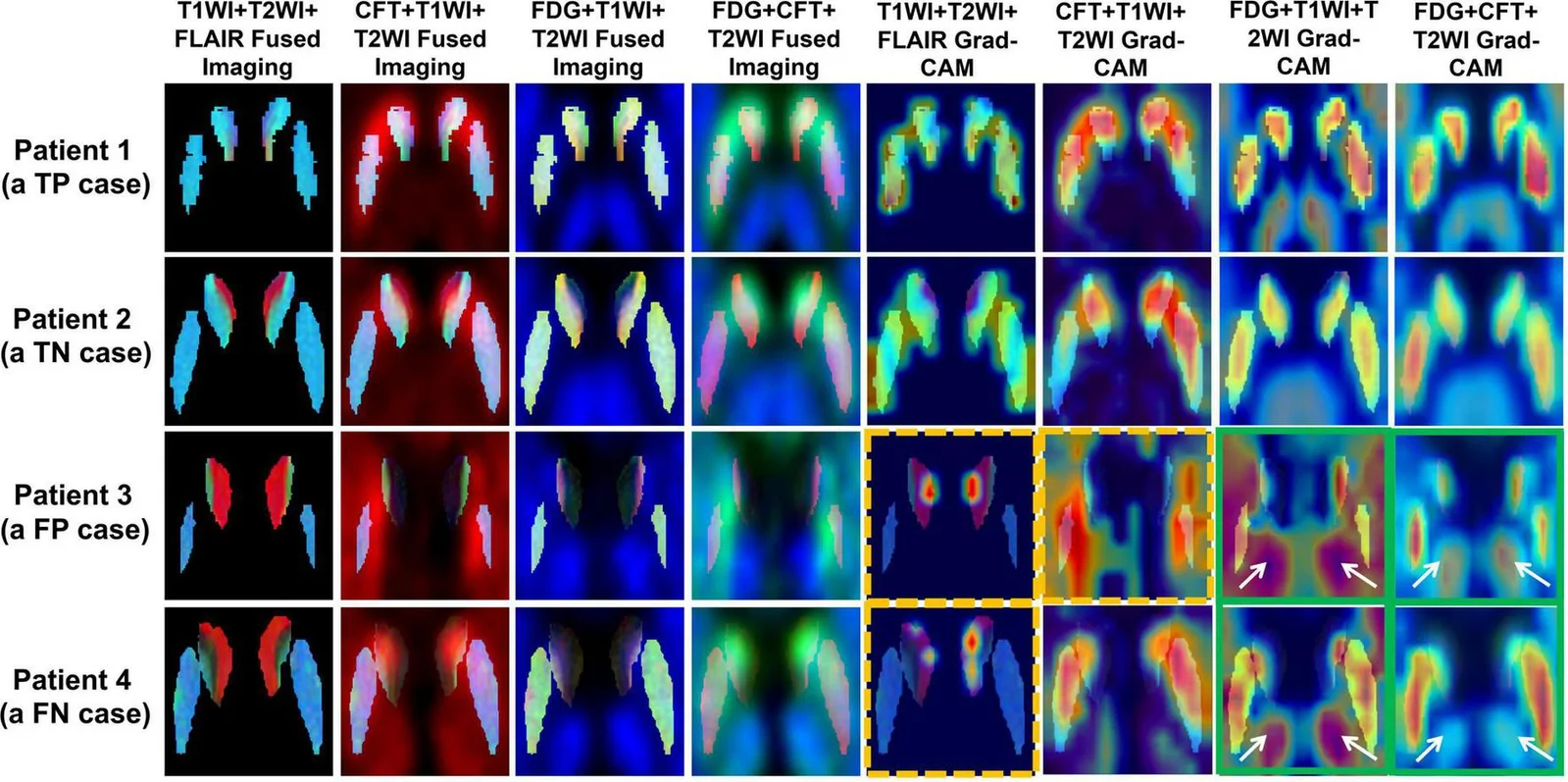
Fused and Grad-CAM visualizations of the Vision Transformer with multi-slice voting (ViT+MSV) model highlighting activation regions across different multimodal input combinations. Each column represents a specific modality combination, and each row corresponds to a different patient. Patients 1 and 2 are correctly classified as multiple system atrophy-parkinsonian type (MSA-P) and Parkinson’s disease (PD), respectively. Patients 3 and 4 represent misclassified cases in T1WI+T2WI+FLAIR: MSA-P misidentified as PD, and PD misidentified as MSA-P, but still correct in CFT+T1WI+T2WI, FDG+T1WI+T2WI, FDG+CFT+T2WI.

In some cases, weaker secondary activation was also observed in adjacent structures, including the thalamus, internal capsule, and periventricular regions (white arrows). As these regions lie close to or are anatomically connected with the striatum within the basal ganglia–thalamocortical motor circuit, this pattern suggests that the model draws predominantly on the putaminal signal while incorporating a limited degree of surrounding anatomical context, rather than relying on isolated or spurious focal features.

To make the slice-to-patient aggregation transparent, Figure 6 presents the five slice-level Grad-CAM maps, PD softmax probabilities, and slice-level predicted labels for four representative held-out cases, together with the resulting weighted MSV score and patient-level decision. The rows illustrate correct and incorrect classifications for both diagnostic groups.

**FIGURE 6:**
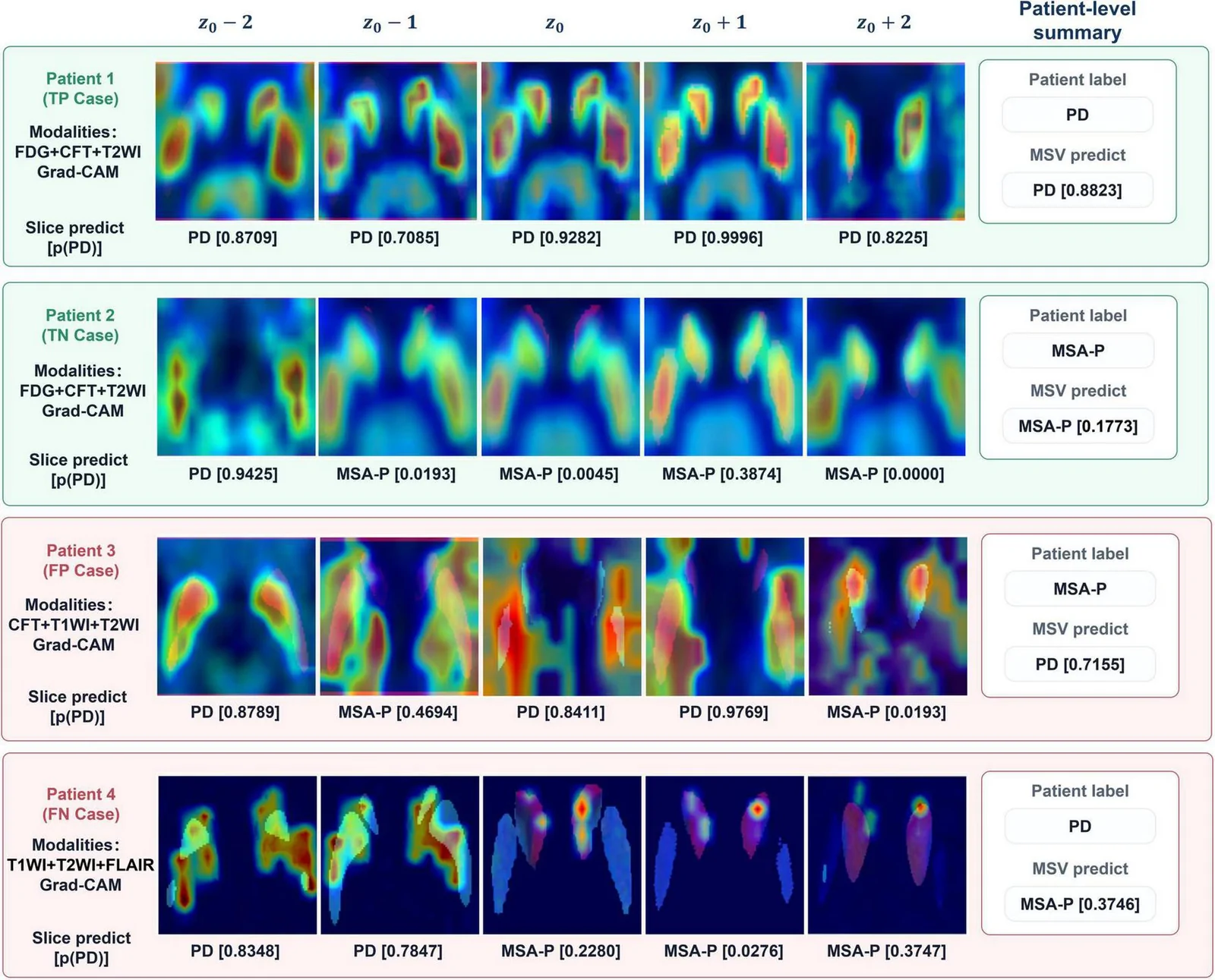
Slice-level gradient attribution and patient-level weighted multi-slice voting in representative cases. Each row shows the same five axial ROI positions from one de-identified patient, together with the slice-level Grad-CAM map, Parkinson’s disease (PD) softmax probability, and predicted label. The patient-level PD probability was calculated using Equation 1. The rows show representative correct and incorrect decisions for PD and multiple system atrophy-parkinsonian type (MSA-P), with PD defined as the positive class. All Grad-CAM maps were generated for the PD class.

## 4 Discussion

This study shows that an anatomically guided multimodal PET/MRI framework can support early differentiation between PD and MSA-P in a clinically challenging cohort (Shokrpour et al., 2025, Fallahpoor et al., 2024). By integrating nnU-Net segmentation and Vision Transformer-based classification, our approach effectively differentiates PD from MSA-P by analyzing complex imaging patterns at early clinical stages.

The systematic comparison of imaging-sequence combinations revealed several patterns that are consistent with the known pathophysiology of early parkinsonism. First, the two PET tracers substantially outperformed all structural MRI sequences when used in isolation (Supplementary Table 1). This aligns with the clinical observation that early-stage PD and MSA-P are primarily distinguished by striatal dopaminergic denervation and metabolic alterations, whereas conventional structural MRI often appears largely unremarkable at this stage, with putaminal and caudate morphometry remaining within normal ranges (Bae et al., 2021; Chen et al., 2022). Second, all top-performing combinations contained both FDG and CFT (FDG+CFT+T2WI 0.947, FDG+CFT+T1WI 0.929, FDG+CFT+FLAIR 0.904). This indicates that metabolic (FDG) and dopaminergic (CFT) information is largely complementary rather than redundant, jointly characterizing distinct aspects of nigrostriatal degeneration. Third, once both PET tracers were included, the choice of the accompanying MRI sequence had a smaller effect on performance than the inclusion of both PET tracers (T2WI ≥ T1WI ≥ FLAIR), suggesting that the dominant classification signal originates from the dual-tracer PET, while MRI provides complementary structural information and the anatomical basis for ROI localization in the present framework. Together, these findings provide an ablation-based, pathophysiologically grounded rationale for the proposed multimodal design, rather than a purely data-driven accumulation of modalities.

Unlike conventional radiomics and other traditional machine learning methods, which depend on predefined, handcrafted features and manual feature selection, the proposed framework learns discriminative representations directly from the fused images in an end-to-end manner (Sun et al., 2024; Chen et al., 2023; Cheng et al., 2025; Huang et al., 2025). This reduces operator-dependent variability and better captures the high-dimensional, heterogeneous patterns present in multimodal PET/MRI data. Consistent with this interpretation, the proposed ViT+MSV framework outperformed the radiomics baseline re-evaluated on the same cohort under the identical cross-validation protocol, supporting the added value of end-to-end learning over handcrafted-feature approaches.

Grad-CAM analysis indicated that class-specific saliency was mainly concentrated in anatomically relevant regions, particularly the bilateral putamen, with occasional secondary activation in adjacent structures such as the thalamus. This pattern suggests that the model relied predominantly on putaminal information while incorporating limited surrounding anatomical context. The activations were largely localized to striatal regions whose anatomical delineation had been assessed in the segmentation analysis, providing visual support for anatomically plausible model sensitivity.

The anatomical restriction of the present pipeline should be considered when interpreting the Grad-CAM maps. Because classification was performed on inputs centered on the caudate nuclei and putamina, the analysis could not assess potentially informative saliency outside this predefined field of view. In a recent whole-brain 3D CNN study using T1-weighted MRI, regional saliency was associated with UPDRS Part III and MoCA scores across nigrostriatal and other subcortical regions, as well as temporal and insular cortices; saliency also covaried within functional networks (Moroze et al., 2025). These findings motivate extending the same multimodal-fusion and multi-slice aggregation framework to broader brain regions and network-level inputs, including structures anatomically and functionally connected to the striatum within basal-ganglia and nigrostriatal circuits, and directly comparing ROI-restricted and whole-brain models in independent cohorts. Importantly, such saliency should be interpreted as model sensitivity rather than direct evidence of causal neuropathology.

Despite promising outcomes, several limitations remain. First, the sample size is relatively small and class distribution was imbalanced, which may have influenced class-specific performance estimates despite the use of stratified cross-validation. Second, some unconventional MRI sequences (e.g., SWI, DWI), although potentially useful for distinguishing PD and MSA, were excluded due to their limited availability in routine clinical practice, as our goal was to identify discriminative features from sequences that are widely acquired. Third, our cohort included only the parkinsonian subtype of MSA (MSA-P); the cerebellar subtype (MSA-C) and other atypical parkinsonian syndromes such as progressive supranuclear palsy and corticobasal degeneration were not evaluated, and the applicability of the proposed framework to these conditions remains to be established in future work. Fourth, the single-tracer PET experiments were not fully MRI independent because anatomical localization and slice selection still relied on MRI-derived striatal segmentation. Therefore, the present study does not establish a stand-alone PET-only workflow. Future work should investigate PET-based or template-based localization and more general missing-modality strategies, such as modality dropout or cross-modal imputation. In addition, our analysis focused on the dorsal striatum (caudate nucleus and putamen), which carries the principal discriminative signal for motor parkinsonism. The ventral striatum, including the nucleus accumbens, was not separately delineated; although its involvement in early PD and MSA-P is less established, ventral striatal dopaminergic and metabolic changes may carry complementary diagnostic information. Future studies should include these additional anatomical structures to improve diagnostic coverage of MSA subtypes (Krismer et al., 2024). Finally, although Grad-CAM provides spatially grounded visual interpretability, gradient-based saliency visualization captures only part of the decision-making process of deep learning models. Future work integrating complementary explainability approaches—such as feature attribution or concept-based analysis—could further enhance model transparency and support broader clinical adoption.

## 5 Conclusion

In summary, we propose a fully automated, interpretable PET/MRI deep learning framework that integrates atlas-guided segmentation with Vision Transformer–based classification for the early differentiation of PD and MSA-P. On a cohort of 155 early-stage patients evaluated by 4-fold cross-validation, the framework achieved an AUC of 0.947 and outperformed both deep learning baselines and a conventional radiomics approach, with Grad-CAM showing that class-specific saliency was predominantly concentrated in anatomically relevant striatal regions. These results suggest that the framework may serve as a practical decision-support tool for early differential diagnosis, while external multi-center validation and extension to other parkinsonian syndromes remain important directions for future work.

## Data Availability

The raw data supporting the conclusions of this article will be made available by the authors, without undue reservation.

## Footnotes

1
http://Neuromorphometrics.com

2
https://slicer.org/

3
https://www.fil.ion.ucl.ac.uk/spm/software/spm12/
