# Supplementary material for: Differentiating early Parkinson’s disease from multiple system atrophy on multimodal PET/MRI using atlas-guided segmentation and vision transformer classification
Source: Front Aging Neurosci. 2026 Sep 25;18:1928959. doi: 10.3389/fnagi.2026.1928959 (PMC13649701; doi:10.3389/fnagi.2026.1928959)
Supplement: Supplementary file 1 [file Table_1.docx]

Supplemental Tables

**Table S1**. Cross-validated performance of all evaluated single- and three-modality imaging sequence combinations

| **Modality(s)** | **RGB channel order**  **R/G/B** | **AUC**  **[95% CI]** | **ACC**  **[95% CI]** | **Sens**  **[95% CI]** | **Spec**  **[95% CI]** |
| --- | --- | --- | --- | --- | --- |
| **T1WI** | T1WI/T1WI/T1WI | 0.760  [0.663-0.849] | 0.774  [0.710-0.839] | 0.769  [0.695-0.842] | 0.794  [0.645-0.923] |
| **T2WI** | T2WI/T2WI/T2WI | 0.723  [0.625-0.819] | 0.742  [0.671-0.813] | 0.727  [0.646-0.803] | 0.794  [0.641-0.923] |
| **FLAIR** | FLAIR/FLAIR/FLAIR | 0.698  [0.581-0.805] | 0.697 [0.626-0.768] | 0.669  [0.587-0.752] | 0.794  [0.645-0.923] |
| **CFT** | CFT/CFT/CFT | 0.840  [0.752-0.917] | 0.858  [0.800-0.910] | 0.876  [0.816-0.934] | 0.794  [0.645-0.923] |
| **FDG** | FDG/FDG/FDG | 0.859 [0.789-0.925] | 0.858 [0.800-0.910] | 0.868 [0.805-0.926] | 0.824  [0.684-0.946] |
| **T1WI+T2WI+ FLAIR** | T2WI/T1WI/FLAIR | 0.764  [0.674-0.843] | 0.787  [0.723-0.845] | 0.785  [0.711-0.854] | 0.794  [0.645-0.923] |
| **CFT+T1WI+ FLAIR** | CFT/FLAIR/T1WI | 0.830  [0.755-0.898] | 0.858  [0.800-0.910] | 0.851  [0.785-0.911] | 0.882  [0.759-0.973] |
| **FDG+T1WI+ FLAIR** | FLAIR/T1WI/FDG | 0.825  [0.742-0.898] | 0.845  [0.787-0.897] | 0.826  [0.754-0.890] | 0.912  [0.800-1.000] |
| **CFT+T2WI+ FLAIR** | FLAIR/CFT/T2WI | 0.814  [0.721-0.894] | 0.858  [0.800-0.910] | 0.860  [0.794-0.919] | 0.853  [0.722-0.969] |
| **FDG+T2WI+ FLAIR** | FLAIR/T2WI/FDG | 0.859  [0.797-0.914] | 0.832  [0.768-0.890] | 0.810 [0.738-0.879] | 0.912  [0.809-1.000] |
| **CFT+T1WI+T2WI** | T2WI/T1WI/CFT | 0.832  [0.750-0.906] | 0.858  [0.800-0.910] | 0.860  [0.795-0.917] | 0.853  [0.722-0.969] |
| **FDG+T1WI+T2WI** | T2WI/T1WI/FDG | 0.884  [0.817-0.942] | 0.884  [0.832-0.929] | 0.884  [0.825-0.937] | 0.882  [0.765-0.974] |
| **FDG+CFT+ FLAIR** | FLAIR/CFT/FDG | 0.904  [0.831-0.964] | 0.929  [0.884-0.968] | 0.934  [0.884-0.975] | 0.912  [0.800-1.000] |
| **FDG+CFT+**  **T1WI** | T1WI/CFT/FDG | 0.929  [0.879-0.971] | 0.929  [0.884-0.968] | 0.926  [0.875-0.968] | 0.941  [0.853-1.000] |
| **FDG+CFT+**  **T2WI** | T2WI/CFT/FDG | 0.947  [0.905-0.981] | 0.929  [0.890-0.968] | 0.942  [0.900-0.982] | 0.882  [0.765-0.974] |
